# The Clinical Significance of Serum Sirtuin 2 in Diabetic Nephropathy: Evidence for a Potential Biomarker of Renal Injury

**DOI:** 10.3390/jcm14228095

**Published:** 2025-11-15

**Authors:** Ozgur Yilmaz, Osman Erinc, Murvet Algemi, Recep Demirci, Sengul Aydin Yoldemir, Murat Akarsu

**Affiliations:** 1 Department of Internal Medicine, Kanuni Sultan Suleyman Training and Research Hospital, Atakent Distr., Turgut Ozal Blvd., No: 46/1, Kucukcekmece, 34303 Istanbul, Turkey; doctorerinc@gmail.com (O.E.);; 2 Department of Clinical Biochemistry, Kanuni Sultan Suleyman Training and Research Hospital, 34303 Istanbul, Turkey; algemimurvet@gmail.com; 3 Department of Nephrology, Kanuni Sultan Suleyman Training and Research Hospital, 34303 Istanbul, Turkey

**Keywords:** Sirtuin 2 (SIRT2), diabetic nephropathy, type 2 diabetes mellitus, biomarker, microvascular complications

## Abstract

**Background/Objectives:** Type 2 diabetes mellitus (T2DM) is a major metabolic disorder associated with progressive microvascular complications such as nephropathy, retinopathy, and neuropathy. Early detection of diabetic nephropathy (DN) remains challenging, as conventional markers such as urine albumin-to-creatinine ratio (UACR) and estimated glomerular filtration rate (eGFR) are influenced by non-renal factors and lack sensitivity for subclinical injury. Sirtuin 2 (SIRT2), a cytoplasmic NAD^+^-dependent deacetylase involved in oxidative stress and inflammatory regulation, has recently been implicated in renal pathophysiology. This study aimed to assess the relationship between serum SIRT2 levels and the presence of diabetic nephropathy and to evaluate its potential utility as a complementary biomarker reflecting early renal injury. **Methods:** In this single-center, cross-sectional study, 180 participants aged 18–80 years were enrolled: 60 healthy controls, 60 T2DM patients without nephropathy (T2DM − DN), and 60 T2DM patients with nephropathy (T2DM + DN). Serum SIRT2 concentrations were quantified using a validated ELISA. Group comparisons, multinomial logistic regression, and receiver operating characteristic (ROC) curve analyses were performed to assess associations between SIRT2 and renal indices (UACR and eGFR). Statistical significance was set at *p* < 0.05. **Results:** Serum SIRT2 concentrations showed a progressive elevation across study groups (*p* < 0.001), with median levels of 6.13 ng/mL in healthy controls, 8.53 ng/mL in T2DM − DN, and 33.19 ng/mL in T2DM + DN. ROC analysis revealed good diagnostic performance for differentiating DN from healthy controls (AUC = 0.813, sensitivity 75%, and specificity 78.3%). Multivariable regression analysis identified SIRT2 as an independent correlate of DN after adjusting for metabolic and renal covariates (adjusted OR = 1.22, 95% CI 1.11–1.35, *p* < 0.001). **Conclusions:** Serum SIRT2 levels were observed to increase in parallel with the presence and severity of diabetic nephropathy and remained independently associated with the condition after adjustment for conventional risk factors. These findings suggest that SIRT2 may serve as a feasible complementary biomarker reflecting renal injury processes not captured by traditional measures. Further longitudinal studies are warranted to clarify its prognostic significance and potential for clinical integration.

## 1. Introduction

Diabetic nephropathy (DN) remains one of the most severe microvascular complications of type 2 diabetes mellitus (T2DM), contributing substantially to end-stage renal disease (ESRD) and accounting for nearly half of incident dialysis cases worldwide [1]. Despite notable improvements in glycemic and blood-pressure control, the global burden of diabetic nephropathy keeps increasing, highlighting the need for novel, sensitive, and specific biomarkers for early diagnosis and risk assessment [2,3]. DN often evolves insidiously over years, with early tissue damage occurring before clinical signs and hindering timely recognition. Although clinical evaluation primarily depends on albuminuria and estimated glomerular filtration rate (eGFR), a considerable subset of individuals with T2DM develop kidney damage without increased urinary albumin (non-albuminuric DN) [4,5]; thus, albuminuria may be absent even in histologically confirmed disease, and a decline in eGFR usually only appears after appreciable nephron loss [6,7].

Urine albumin–creatinine ratio (UACR) and eGFR are influenced by non-renal factors and often fail to detect early kidney injury, which limits their specificity for active kidney damage [8]. Recent evidence further indicates that these traditional markers also lack sufficient sensitivity to identify early diabetic kidney disease stages, emphasizing the importance of developing novel and mechanism-based biomarkers [9]. Serum-based biomarkers with higher reproducibility, superior stability, and suitability for repeated testing may better reflect subclinical disease activity; a circulating marker that captures metabolic and inflammatory signals could improve risk assessment beyond UACR and eGFR [10].

The sirtuin family (SIRT1–SIRT7) comprises NAD^+^-dependent deacetylases that regulate cellular responses to metabolic and oxidative stress, such as energy metabolism, mitochondrial maintenance, autophagy, and inflammatory signaling [11,12]. In the kidney under diabetic conditions, several sirtuins have been shown in experimental and preclinical studies; notably, SIRT1, SIRT3, and SIRT6 have been found to reduce oxidative damage, lessen profibrotic TGF-β/SMAD activity, and enhance autophagy-mediated quality control [9,13,14]. Beyond renal tissue, sirtuins are involved in regulating systemic glucose and lipid regulation, insulin sensitivity, vascular function, and mitochondrial adaptation in diabetes, providing a biological context for their potential relevance to diabetic kidney disease [11,12,15].

Among aberrant sirtuin family members, Sirtuin 2 (SIRT2) has emerged as a less explored but potentially pivotal factor in DN pathophysiology. SIRT2 is primarily localized in the cytoplasm and is abundantly expressed in renal tubular epithelial cells [16]. Experimental studies have demonstrated that SIRT2 enhances insulin sensitivity and glucose uptake by deacetylating FOXO1 and interacting with PGC-1α, thereby influencing adipocyte differentiation and hepatic energy metabolism [17,18]. Additionally, SIRT2 helps maintain redox homeostasis by deacetylating glucose-6-phosphate dehydrogenase (G6PD), supporting NADPH production and antioxidant defenses [19]. Furthermore, acetylation of phosphoenolpyruvate carboxykinase 1 promotes its degradation via ubiquitin-mediated pathways, whereas deacetylation enhances its stability, thereby increasing gluconeogenic flux. Although SIRT2 was not directly implicated in this study, its known deacetylase activity on metabolic enzymes suggests a potential role in modulating hepatic gluconeogenesis through similar pathways [20]. Collectively, these findings position SIRT2 at the intersection of metabolic and inflammatory pathways that are central to the pathogenesis of diabetic complications [21].

Preclinical models further suggest that SIRT2 has a complex and context-specific role in kidney disease. In diabetic mice and tubular cells exposed to high glucose, increased SIRT2 levels are associated with the activation of pro-inflammatory gene programs, macrophage recruitment, and worsened tubular damage [22,23]. Conversely, in non-diabetic models of renal fibrosis, SIRT2 exhibits antifibrotic effects by modulating TGF-β/SMAD signaling pathways [24,25]. This dichotomy underscores the biological complexity of SIRT2 and emphasizes the importance of defining its role specifically within diabetic kidney disease [26].

Clinical evidence on SIRT2 in human DN remains extremely limited. One recent study demonstrated a correlation between urinary SIRT2 levels and tubular injury in T2DM, suggesting translational relevance [27]. However, serum SIRT2, which offers distinct advantages in terms of accessibility, stability, and feasibility for repeated monitoring, has not been investigated to date [28,29]. Accordingly, to the best of our knowledge based on a comprehensive literature search, the present study is the first to quantify serum SIRT2 in DN and examine its associations with established renal indices.

This study was designed to investigate serum SIRT2 concentrations in the context of diabetic nephropathy by comparing patients with T2DM with and without nephropathy to healthy controls, and by assessing their relationships with established renal markers such as eGFR and UACR in order to determine whether SIRT2 may serve as a clinically meaningful biomarker that reflects renal injury beyond conventional indices and supports early detection and risk stratification.

## 2. Materials and Methods

### 2.1. Study Design and Setting

This single-center, cross-sectional study included 180 participants, aged between 18 and 80 years old, comprising 120 patients diagnosed with T2DM and 60 healthy controls, all recruited from the internal medicine outpatient clinics of our hospital. Participants were equally divided into three groups (*n* = 60 each): Group 1, healthy control subjects without diabetes; Group 2, patients with T2DM but without DN; and Group 3, patients with T2DM and DN, including both microalbuminuric and macroalbuminuric cases. To minimize confounding, the groups were matched based on age and sex. The diagnosis of T2DM was established according to the 2024 American Diabetes Association Standards of Care [30]. Standard glycemic criteria were applied, and any abnormal test result was reconfirmed on a separate day, unless patients presented with unequivocal hyperglycemia accompanied by classic symptoms. The diagnosis of DN followed the Kidney Disease: Improving Global Outcomes (KDIGO) 2023 Clinical Practice Guideline [31]. DN was identified as a UACR of ≥30 mg/g in at least two of three measurements over three months and/or an eGFR less than 60 mL/min/1.73 m^2^, calculated with the CKD-EPI equation. Microalbuminuria was defined as UACR 30–300 mg/g, while macroalbuminuria was defined as UACR > 300 mg/g. All biochemical measurements were repeated on two separate occasions to ensure diagnostic reliability. The sample size was determined using G*Power 3.1.9.2. Based on preliminary data from the first 30 participants in each group, the effect size for one-way ANOVA (fixed effects) was calculated as Cohen’s f = 0.31. Using α = 0.05, power (1 − β) = 0.95, and three groups, the minimum required sample size was 164 participants. To ensure adequate statistical power, 60 individuals were included per group (*n* = 180). A post hoc power analysis confirmed that this sample size provided >80% power to detect the observed difference in serum SIRT2 levels.

### 2.2. Exclusion Criteria

To ensure a homogeneous study population and minimize confounding, participants were excluded if they had type 1 diabetes, acute metabolic decompensation (e.g., diabetic ketoacidosis or hyperosmolar state), or evidence of non-diabetic kidney disease (e.g., glomerulonephritis, polycystic kidney disease, obstructive uropathy, recent acute kidney injury, or renal replacement therapy). Additional exclusions included active or recent malignancy, advanced liver disease, chronic infections, autoimmune or systemic inflammatory disorders, untreated thyroid dysfunction, chronic corticosteroid or immunosuppressive therapy, pregnancy or lactation, organ transplantation, or inability to provide informed consent.

### 2.3. Data Collection

Demographic and clinical information, including age, sex, family history, smoking status, and duration of T2DM, was recorded. The control group consisted of age- and sex-matched healthy volunteers recruited from the same hospital population. All controls had normal fasting plasma glucose (FPG), eGFR > 90 mL/min/1.73 m^2^, normoalbuminuria, and no history of diabetes, hypertension, dyslipidemia, or other systemic diseases. Smoking status was recorded as non-smoker, former smoker, or active smoker. BMI was determined as weight (kg)/height (m^2^). After a brief rest, right arm systolic and diastolic blood pressures were recorded in the sitting position and the average of two consecutive measurements obtained at five-minute intervals was used for analysis. Information on the use of antidiabetic, antihypertensive, and lipid-lowering medications was collected from patient interviews and verified against medical records.

### 2.4. Blood Sampling and Biochemical Analysis

Fasting venous blood tests were obtained between 8:00 a.m. and 10:00 a.m. following a 10 to 12 h fasting period. Blood was drawn from the brachial vein in the antecubital fossa into plain, anticoagulant-free tubes for serum analysis. Laboratory staff conducting the assays were blinded to participants’ group allocation to prevent measurement bias. Samples were centrifuged at 4 °C for 10 min at 4000 rpm, and biochemical analyses were performed immediately on freshly separated serum. FPG, serum creatinine, alanine aminotransferase (ALT), albumin, and lipid profile (total cholesterol, triglyceride, high-density lipoprotein cholesterol (HDLc), and low-density lipoprotein cholesterol (LDLc)) were determined using enzymatic colorimetric methods on the Roche Cobas 8000 c702 analyzer (Roche Diagnostics, Mannheim, Germany). C-reactive protein (CRP) levels were measured via an immunoturbidimetric assay on the same analyzer. Glycated hemoglobin (HbA1c) was assessed using high-performance liquid chromatography with the ARKRAY/ADAMS HA-8180V system (ARKRAY Inc., Kyoto, Japan). Plasma aliquots designated for additional analyses were promptly frozen and stored at −80 °C until further evaluation.

### 2.5. Measurement of Serum Sirtuin 2 Level

Serum SIRT2 concentrations were determined as the primary biomarker in this study. Following an overnight fast of 10–12 h, venous blood samples were collected from the antecubital vein, allowed to clot at room temperature for 10–20 min, and centrifuged at 4 °C for 20 min at 2000–3000 rpm. The resulting serum was aliquoted into Eppendorf tubes and stored at −80 °C until analysis, avoiding repeated freeze–thaw cycles. Quantification of SIRT2 was performed using a double-antibody sandwich enzyme-linked immunosorbent assay kit (Human SIRT2 ELISA Kit, Sunred Biological Technology Co., Shanghai, China; Catalog No. 201-12-2559) according to the manufacturer’s protocol. Briefly, 50 μL of standards or 40 μL of serum samples plus 10 μL of biotin-labeled SIRT2 antibody were added to the designated wells, followed by 50 μL of streptavidin HRP solution. Plates were sealed and incubated for 60 min at 37 °C. After incubation, plates were washed five times with diluted wash buffer to remove unbound material. Subsequently, 50 μL of Chromogen Solution A and 50 μL of Chromogen Solution B were added to each well, and the plates were incubated for 10 min at 37 °C protected from light. The reaction was stopped by adding 50 μL of stop solution, and optical density was measured at 450 nm within 15 min using a calibrated microplate reader. The mean values were used for statistical analysis. The assay had a sensitivity of 0.147 ng/mL, with a quantifiable range of 0.2–60 ng/mL. Intra-assay and inter-assay coefficients of variation were <10% and <12%, respectively, indicating high analytical precision. No cross-reactivity with other proteins was reported by the manufacturer. Analytical validation data, including linearity (r > 0.99), recovery (90–110%), and intra/inter-assay precision (<10% and <12%), were provided by the manufacturer. Independent in-house validation was not performed; all samples were analyzed in duplicate to ensure reproducibility.

### 2.6. Outcomes

The primary outcome was to evaluate serum SIRT2 concentrations as a potential biomarker for DN by comparing levels among healthy controls, patients with T2DM without DN, and patients with T2DM and DN. Secondary outcomes included examining the associations between serum SIRT2 levels and disease severity, assessed through albuminuria categories (normo-, micro-, and macroalbuminuria) and eGFR strata.

### 2.7. Statistical Analysis

IBM SPSS Statistics for Windows 26.0 (IBM Corp., Armonk, NY, USA) and Python 3.13 (64-bit) were used for all statistical analyses. In the Python environment, pandas (v2.2.2), numpy (v1.26.4), matplotlib (v3.9.2), seaborn (v0.13.2), and scikit-learn (v1.5.1) libraries were used. The normality of continuous variables was evaluated using the Kolmogorov–Smirnov and Shapiro–Wilk test and homogeneity of variances was assessed with Levene’s test. Data variables with normal distributions were expressed as mean ± standard deviation, whereas non-normally distributed data were presented as median (interquartile range, Q1–Q3). The chi-square test was utilized to compare categorical variables that were shown as frequencies and percentages. Comparisons among three groups were assessed using one-way ANOVA or the Kruskal–Wallis test; pairwise comparisons were performed using the Independent Samples *t*-test or Mann–Whitney U test, as appropriate. Post hoc analyses were conducted using Tukey or Games–Howell tests for parametric data and the Dunn–Bonferroni correction for non-parametric data to control the increased risk of Type I error. Multinomial logistic regression was performed to determine the odds ratios between groups under the influence of age, sex, and body mass index (BMI). The results are presented in forest plots with analytical odds ratios (OR) and 95% confidence intervals (CIs). Serum SIRT2 levels were found to be non-normally distributed, and the values were retested by incorporating the standard deviation increase (based on the standard deviation increase) into multivariate logistic regression models adjusted for age, sex, and body mass index (BMI). Model calibration was assessed using the Hosmer–Lemeshow goodness-of-fit test, and internal validity (internal validation) was assessed using 2000 bootstrap specifications. Receiver operating characteristic (ROC) curves were generated to assess the diagnostic accuracy of serum SIRT2 and other predictors. The area under the curve (AUC) with 95% CIs was calculated, and optimal cut-off values were determined using the Youden index. For each cut-off, sensitivity, specificity, positive predictive value (PPV), and negative predictive value (NPV) were reported. Multicollinearity among variables was assessed using the Variance Inflation Factor (VIF) and Tolerance values; all VIF measurements were below 2.0 and all Tolerance values were above 0.60. In addition, the three biomarkers were also evaluated using a multivariable (combined) ROC analysis. The combined model was constructed based on a multivariable logistic regression model that included all three biomarkers (SIRT2 + UACR + GFR) together. Using a one-vs-rest approach, the discriminatory ability for each group was assessed, and AUC values were calculated with 2000 bootstrap resamples and corresponding 95% confidence intervals. Optimal cut-off values were determined according to the Youden J statistic. Model calibration was evaluated using the Hosmer–Lemeshow test and the Brier score. The discriminative performance of the combined model was compared with that of individual biomarkers using the DeLong test to determine the statistical significance of AUC differences. A *p*-value < 0.05 was considered statistically significant.

## 3. Results

A total of 180 participants were included in the study, comprising 60 healthy controls (HC), 60 patients with type 2 diabetes mellitus without nephropathy (T2DM − N), and 60 patients with diabetic nephropathy (T2DM + N). Within the T2DM + N group, 34 patients (56.7%) had microalbuminuria and 26 patients (43.3%) had macroalbuminuria. Of the entire study population, 92 participants (51.1%) were female and 88 (48.9%) were male. When the study cohort was evaluated according to the KDIGO 2023 Clinical Practice Guideline for chronic kidney disease (CKD), 118 participants (65.6%) were classified as stage G1 (eGFR ≥ 90 mL/min/1.73 m^2^), 40 (22.2%) as stage G2 (60–89 mL/min/1.73 m^2^), 10 (5.6%) as stage G3a (45–59 mL/min/1.73 m^2^), 4 (2.2%) as stage G3b (30–44 mL/min/1.73 m^2^), 3 (1.7%) as stage G4 (15–29 mL/min/1.73 m^2^), and 5 (2.8%) as stage G5 (eGFR < 15 mL/min/1.73 m^2^).

The mean ages were 57.1 ± 8.9 years in the healthy control group, 59.0 ± 9.9 years in the T2DM − N group, and 60.5 ± 10.9 years in the T2DM + N group, with no statistically significant difference among the groups (*p* = 0.182). Age and sex distributions did not differ significantly among the three groups (*p* = 0.182 and *p* = 0.915, respectively). BMI was higher in both diabetic subgroups compared with healthy controls (*p* = 0.019), with the highest values observed in the T2DM + N group. Systolic and diastolic blood pressures were significantly elevated in T2DM − N and T2DM + N patients relative to controls (*p* < 0.001 for both), while no significant difference was observed between the two diabetic subgroups. Among patients with type 2 diabetes mellitus (*n =* 120), 69 (57.5%) were receiving antihypertensive therapy, including angiotensin-converting enzyme (ACE) inhibitors or angiotensin II receptor blockers (ARBs) in 52 (43.3%) patients, calcium channel blockers (CCBs) in 28 (23.3%), beta-adrenergic blockers (beta-blockers) in 18 (15.0%), and diuretics in 14 (11.7%). Regarding antidiabetic therapy, 88 (73.3%) were treated with metformin, 46 (38.3%) with dipeptidyl peptidase-4 (DPP-4) inhibitors, 22 (18.3%) with sodium-glucose cotransporter-2 (SGLT2) inhibitors, 14 (11.7%) with glucagon-like peptide-1 (GLP-1) receptor agonists, and 16 (13.3%) with sulfonylureas or thiazolidinediones, either alone or in combination with other agents. The distribution of these medication classes did not differ significantly between the T2DM − N and T2DM + N groups (*p* > 0.05). Table 1 provides the clinical and demographic data of the study cohort.

As shown in Table 2, FPG, HbA1c, and homeostasis model assessment of insulin resistance (HOMA-IR) were significantly higher in both diabetic groups compared with healthy controls (all *p* < 0.001), with further elevation in patients with nephropathy relative to those without (*p* < 0.001). Blood pressure levels also differed significantly: both systolic and diastolic values were higher in diabetic patients than in controls, with the highest measurements observed in the nephropathy group (*p* < 0.05). Serum creatinine was significantly increased and eGFR markedly reduced in the T2DM + N group compared with the other groups (*p* < 0.001). UACR showed a progressive rise from controls to T2DM − N and was highest in T2DM + N (*p* < 0.001).

**Figure 1:**
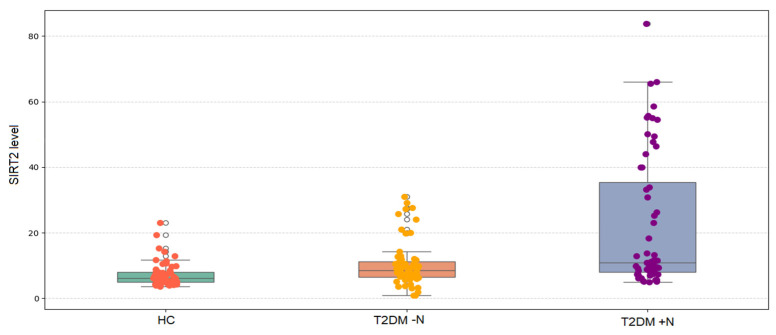
Comparison of SIRT2 levels among healthy controls (HC, *n* = 60), patients with type 2 diabetes mellitus without nephropathy (T2DM − N, *n* = 60), and patients with type 2 diabetes mellitus with nephropathy (T2DM + N, *n* = 60). Box plots display the median (bold line), interquartile range (box), minimum and maximum values (whiskers), and outliers (dots outside the whiskers). Individual data points are overlaid and color-coded.

As shown in Figure 1, serum SIRT2 levels differed significantly among the three groups (Kruskal–Wallis, *p* < 0.001). Median (IQR) values demonstrated a progressive increase, being lowest in healthy controls, higher in patients with T2DM without nephropathy, and highest in those with nephropathy. Post hoc pairwise comparisons with Bonferroni correction confirmed that SIRT2 levels were significantly higher in T2DM − N compared with controls (*p* = 0.007), markedly higher in T2DM + N compared with controls (*p* < 0.001), and significantly higher in T2DM + N compared with T2DM − N (*p* = 0.007).

Median serum SIRT2 levels were 6.13 ng/mL in healthy controls, 8.53 ng/mL in the T2DM − N group, 9.89 ng/mL in the microalbuminuria group, and 33.19 ng/mL in the macroalbuminuria group, with significant differences across groups (*p* < 0.001) (Table 3). Compared with healthy controls, SIRT2 levels were significantly higher in T2DM − N (*p* = 0.014), microalbuminuria (*p* < 0.001), and macroalbuminuria groups (*p* < 0.001). The macroalbuminuria group also showed higher levels than T2DM − N (*p* = 0.033), whereas no significant difference was observed between the microalbuminuria and macroalbuminuria groups.

**Figure 2:**
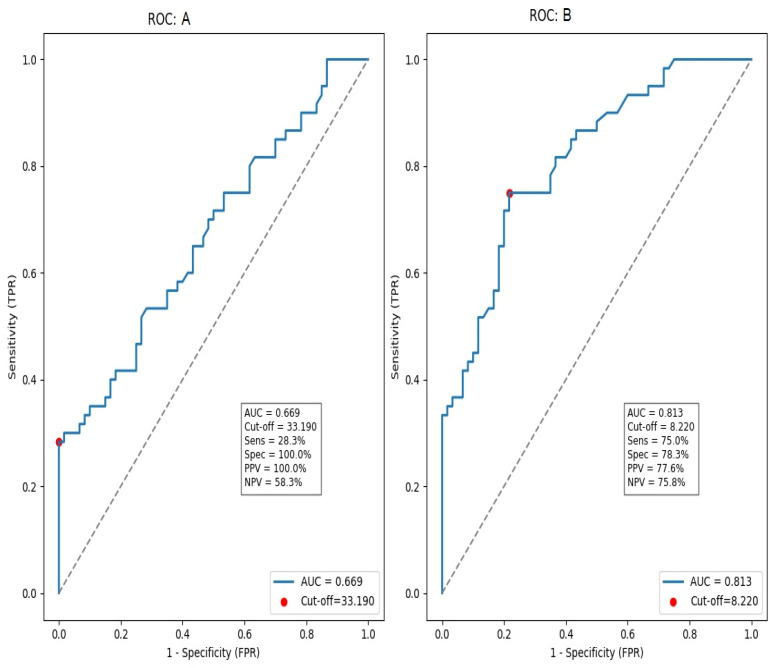
ROC analyses demonstrating the discriminative performance of serum SIRT2 levels across study groups. The blue line indicates the ROC curve, the dashed line represents the line of no discrimination (AUC = 0.5), and the red dot marks the Youden-derived optimal cut-off. ROC A: comparison between T2DM – N (Group 2) and T2DM + N (Group 3). ROC B: comparison between HC (Group 1) and T2DM + N (Group 3). AUC, cut-off, sensitivity, specificity, PPV, and NPV are shown within each graph. AUC: area under the curve; PPV: positive predictive value; NPV: negative predictive value.

ROC analysis showed discrimination between diabetic nephropathy and healthy controls (AUC = 0.813, 95% CI 0.732–0.894; *p* < 0.001). With a Youden-derived cut-off of 8.22 ng/mL, serum SIRT2 achieved 75.0% sensitivity and 78.3% specificity (PPV 77.6%, NPV 75.8%). In contrast, discrimination between people with diabetes with and without nephropathy was modest (AUC = 0.669, 95% CI 0.578–0.761; *p* < 0.001); for this comparison, a cut-off of 33.19 ng/mL yielded 38.3% sensitivity and 100.0% specificity (PPV 100.0%, NPV 59.3%) (Figure 2).

**Figure 3:**
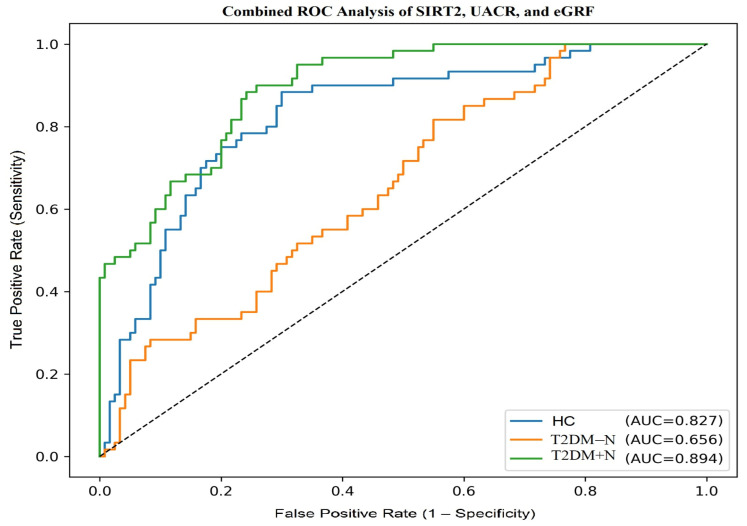
Combined ROC curves of SIRT2, UACR and -eGFR across the three study groups. The ROC curves were generated from a multivariable logistic regression model incorporating SIRT2, UACR and reversed eGFR (−eGFR). A one-vs-rest approach was applied to evaluate the discriminative ability of the model for each group separately. The diagonal dashed line represents the line of no discrimination (random classification). HC: healthy control; T2DM − N: type 2 diabetes mellitus without nephropathy; T2DM + N: type 2 diabetes mellitus with nephropathy.

A combined logistic regression model incorporating SIRT2, UACR and −eGFR was developed to evaluate their joint diagnostic performance. In one-vs-rest ROC analysis, the model yielded AUC values of 0.827 for HC, 0.656 for T2DM − N and 0.894 for T2DM + N. According to DeLong’s test, the combined model demonstrated significantly higher AUC values compared to SIRT2 alone across all groups (*p* < 0.001). Calibration was acceptable in both diabetic groups, and overall prediction accuracy was supported by Brier scores. Optimal cut-off values with corresponding sensitivity and specificity are presented in Table 4, while the ROC curves of the multivariable model are illustrated in Figure 3.

**Figure 4:**
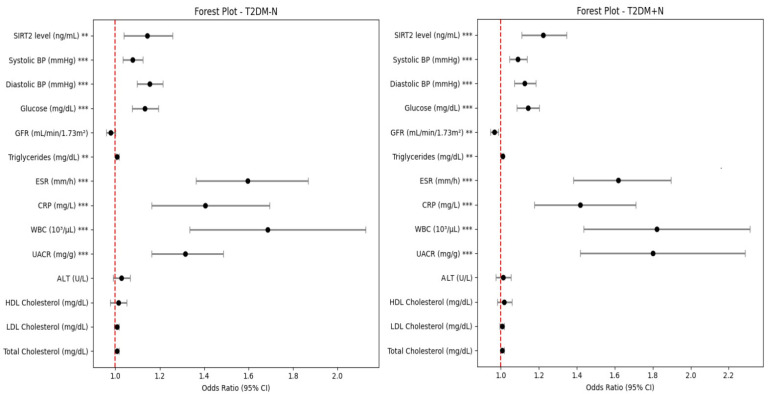
Forest plot of multinomial logistic regression-derived odds ratios. Forest plot displaying adjusted odds ratios (ORs) and 95% confidence intervals from multinomial logistic regression comparing patients with type 2 diabetes without nephropathy (T2DM − N) and with nephropathy (T2DM + N) against healthy controls. Models are adjusted for age, sex, and BMI. Points represent ORs (per-unit increase as specified in Methods) with 95% CIs; the vertical red dashed line denotes the null value OR = 1 (no effect). Variables are presented with ORs and 95% confidence intervals. Statistical significance is denoted by asterisks (** *p* < 0.01; *** *p* < 0.001).

Multinomial logistic regression analyses adjusted for age, sex, and BMI showed that serum SIRT2 was an independent predictor most pronounced in the nephropathy group (T2DM + N: β = 0.20, OR = 1.222, 95% CI 1.109–1.346, *p* < 0.001) and also in patients with type 2 diabetes without nephropathy (T2DM − N: β = 0.13, OR = 1.143, 95% CI 1.039–1.258, *p* = 0.006); as shown in the forest plot (Figure 4), the SIRT2 odds ratios and their 95% confidence intervals lie entirely to the right of the OR = 1 reference line in both comparisons, indicating a statistically significant positive association. eGFR demonstrated a protective association that was significant in the nephropathy group (OR = 0.965, 95% CI 0.944–0.986, *p* = 0.001) and borderline in T2DM − N (*p* = 0.080). Higher systolic and diastolic blood pressures and glucose were consistently associated with increased odds (all *p* < 0.001). Triglyceride, UACR, and inflammatory markers (ESR, CRP and leukocyte) were also significant (all *p* < 0.001), whereas ALT, HDL-C, LDL-C, and total cholesterol were not (all *p* > 0.05). Additionally, because SIRT2 levels were not normally distributed across the study groups, the analyses were repeated using both logarithmic transformation and per–standard deviation (SD) increments. After adjustment for age, sex, and body mass index, each 1-SD increase in SIRT2 was associated with a 2.21-fold higher odds of diabetes without nephropathy compared with controls (OR = 2.205; 95% CI: 1.40–5.60; Hosmer–Lemeshow χ^2^ = 13.871, *p* = 0.085), while in the diabetic nephropathy group this increase was approximately 36.41-fold (OR = 36.413; 95% CI: 7.53–7205.02; Hosmer–Lemeshow χ^2^ = 7.631, *p* = 0.470). Model calibration was adequate in both models as Hosmer–Lemeshow *p*-values were >0.05.

To ensure the validity of these regression models and multicollinearity and β-coefficient metrics were further evaluated. Variance inflation factor (VIF) and Tolerance values demonstrated no evidence of collinearity among the covariates (Age: VIF 1.007, Tolerance 0.993; Sex: VIF 1.005, Tolerance 0.995; BMI: VIF 1.003, Tolerance 0.997), and all biochemical variables exhibited VIF < 2.0 and Tolerance > 0.60. When β-coefficients were examined independently of odds ratios, systolic blood pressure (T2DM − N: β = 0.08; T2DM + N: β = 0.09), diastolic blood pressure (T2DM − N: β = 0.14; T2DM + N: β = 0.12) and fasting plasma glucose (T2DM − N: β = 0.13; T2DM + N: β = 0.13) remained positively associated with nephropathy status. Estimated GFR retained a negative β-coefficient particularly in the nephropathy group (T2DM + N: β = −0.04), while it was borderline in T2DM − N (β = −0.02). Triglyceride (β = 0.01), UACR (β 0.27–0.59), and inflammatory indices including ESR (β 0.47–0.48), CRP (β 0.34–0.35), and leukocyte count (β 0.52–0.60) also remained consistently associated across both diabetic subgroups.

## 4. Discussion

The present study demonstrated that serum SIRT2 levels were highest in patients with diabetic nephropathy, significantly exceeding those observed in both healthy controls and patients with type 2 diabetes without nephropathy. The fact that SIRT2 was not only elevated in diabetes but further increased in the nephropathy group indicates that this biomarker is specifically associated with renal involvement rather than diabetes alone. These results align with the findings of Dai et al. [27], who showed that urinary SIRT2 levels increased in proportion to renal injury in diabetic patients, and Chen et al. [22], who reported in experimental models that SIRT2 overexpression aggravated tubular inflammation and structural damage. Taken together, these clinical and preclinical observations implicate SIRT2 in pathways relevant to diabetic kidney disease, supporting its potential as a nephropathy-related biomarker that complements, rather than replaces, conventional indices.

Subgroup analyses revealed that SIRT2 levels were found to be significantly higher in both microalbuminuric and macroalbuminuric patients compared to patients with type 2 diabetes without nephropathy, with higher levels observed in the macroalbuminuric group. Although the difference between microalbuminuric and macroalbuminuric groups did not reach statistical significance in this cohort, the upward trend suggests a progressive rise with disease severity. This observation is clinically relevant, as it indicates that SIRT2 might serve as an indicator of subclinical renal injury, particularly in early disease stages. MacIsaac et al. [32] previously emphasized that many patients with diabetic kidney disease lack elevated albuminuria despite histological kidney damage. Our findings suggest that SIRT2 could fill this diagnostic gap, capturing injury processes that are not reflected by conventional markers.

In addition to SIRT2, several classical clinical and biochemical parameters differed significantly among the three groups, further supporting the validity of our cohort and highlighting established risk factors for nephropathy. Patients with nephropathy had substantially higher FPG and HbA1c compared with both patients with type 2 diabetes without nephropathy and healthy controls. This finding aligns with the central role of poor glycemic control in microvascular disease risk, also supported by contemporary guidelines on managing diabetes in patients with impaired renal function [33]. Notably, a recent study in type 2 diabetic patients reported that urinary SIRT2 levels correlate with markers of kidney injury, supporting the notion that SIRT2 elevation may occur in the context of, but independently from, classical risk factors [27].

ROC curve analysis revealed important insights into the diagnostic performance of SIRT2. In distinguishing patients with diabetic nephropathy from healthy controls, the AUC was 0.813, indicating good accuracy. At a cut-off value of 8.2 ng/mL, SIRT2 achieved 75% sensitivity and 78% specificity, showing that it can reliably discriminate nephropathy from non-diabetic individuals. When comparing patients with type 2 diabetes with nephropathy to those without, the AUC was lower at 0.669, reflecting moderate accuracy; however, this level of performance is similar to other individual biomarkers of diabetic nephropathy, such as NGAL, KIM-1, and soluble TNF receptors, which typically yield AUC values in the 0.65–0.80 range [34,35]. Importantly, in this comparison, high SIRT2 values identified nephropathy patients with 100% specificity, indicating that markedly elevated concentrations strongly predict the presence of advanced disease. In this study, SIRT2 exhibited meaningful diagnostic performance on its own; however, when combined with UACR and eGFR, its discriminative ability increased further, and this improvement was statistically validated using DeLong’s test. Although SIRT2 alone may not entirely distinguish between diabetic subgroups, its diagnostic power matches that of other recognized biomarkers and can be strengthened when evaluated with traditional renal parameters. This is supported by studies from Harkin et al. [29] and Nielsen et al. [34], which demonstrated that combining multiple markers significantly improves predictive performance, emphasizing SIRT2’s value in integrated diagnostic approaches.

Multinomial logistic regression analysis, visualized with a forest plot, confirmed SIRT2’s independent association with diabetic nephropathy, adjusting for factors like age, sex, BMI, glycemic indices, lipid profile, blood pressure, and renal function. The adjusted odds ratio of approximately 1.22 per unit increase in serum SIRT2 highlights its strong and consistent predictive value across models. In contrast, lipid measures such as LDLc and HDLc lost significance after adjustment, consistent with the findings of Navaneethan et al. [36], who demonstrated that dyslipidemia exerts a weaker effect on kidney outcomes once glycemia and blood pressure are accounted for. Meanwhile, inflammatory markers such as CRP and leukocyte count also retained predictive value, supporting the role of systemic inflammation in diabetic kidney disease pathogenesis. This observation is consistent with recent clinical updates highlighting the critical interplay between chronic low-grade inflammation and oxidative stress in diabetic nephropathy [37].

The forest plot visualization emphasized that SIRT2 had one of the most robust effect sizes, with narrow confidence intervals, which suggests the high reliability of this association. These findings are in line with the proteomic analysis of Zhang et al. [26], who identified SIRT2 as one of the most significantly upregulated proteins in diabetic nephropathy, and with Chen et al. [22], who demonstrated causal effects of SIRT2 overexpression in promoting renal injury in experimental models. Collectively, these results position SIRT2 as a novel, independent biomarker that is complementary to traditional metabolic risk factors and support its potential role in risk stratification of diabetic nephropathy.

This study has several limitations that should be considered. Its cross-sectional design prevents conclusions about causality, making it uncertain whether elevated SIRT2 actively contributes to the development of nephropathy or simply reflects existing renal injury. The research was conducted in a single center with a modest sample size, which may restrict the generalizability of the results and may also explain why the difference between micro- and macroalbuminuric subgroups did not reach statistical significance. Although this was a single-center study involving a regionally homogeneous population, which may limit external validity, methodological rigor was maintained through strict inclusion and exclusion criteria, matched controls, and standardized laboratory procedures. Therefore, the findings are applicable to similar clinical populations, while generalization to other ethnic or geographic groups should be approached with caution. Another limitation is that nephropathy was diagnosed based on clinical and laboratory criteria rather than renal biopsy, which, although widely accepted, does not provide histological confirmation. Circulating SIRT2 levels could also have been influenced by unmeasured confounders, including medication use, dietary factors, and the presence of other diabetic complications such as retinopathy or neuropathy, which were not systematically evaluated. In addition, the absence of longitudinal follow-up data means we cannot determine whether baseline SIRT2 predicts disease progression over time. Despite these limitations, the findings may provide a strong rationale for future multicenter prospective studies incorporating serial SIRT2 measurements and mechanistic investigations to clarify its role as a biomarker of renal injury and to determine whether it is involved in potentially modifiable pathophysiological pathways in diabetic nephropathy.

In conclusion, this study demonstrates that circulating SIRT2 is markedly elevated in patients with diabetic nephropathy, including those at the microalbuminuric stage, and remains independently associated with the disease beyond established risk factors. The stepwise increase observed across study groups, supported by experimental and proteomic findings, suggests that SIRT2 may represent a promising biomarker and could be involved in potentially modifiable pathophysiological pathways in diabetic kidney disease.

## Figures and Tables

**Table 1:** Comparison of baseline demographic and clinical features among groups.

Parameters	Healthy Control (*n* = 60)	T2DM − N *(n =* 60)	T2DM + N *(n =* 60)	*p*
Age, years	57.1 ± 8.9	59 ± 9.9	60 ± 10.9	0.182 ^§^
Sex, *n* (%)				
Female	32 (53.3)	30 (50)	30 (50)	0.915 ^†^
Male	28 (46.7)	30 (50)	30 (50)	
BMI, kg/m^2^	27.89 ± 3.80	29.47 ± 5.72	30.31 ± 4.38	0.019 ^§a^
Systolic BP, mmHg	119.82 ± 6.86	127.9 ± 12.46	129.6 ± 12.34	<0.001 ^§b^
Diastolic BP, mmHg	71.07 ± 5.98	83.30 ± 10.74	81.78 ± 10.87	<0.001 ^§b^
Diabetes duration, years		10 (5–15)	12 (8–15)	0.068 ^t^
Hypertension, *n* (%)	-	34 (56.7)	40 (66.7)	0.260 ^†^
Hyperlipidemia, *n* (%)	-	30 (48.3)	31 (51.7)	0.855 ^†^
Smoking status, *n* (%)				
Never	60 (100)	27 (45)	16 (26.7)	<0.001 ^†^
Ex-smoker	0 (0)	6 (10)	12 (20)	
Current smoker	0 (0)	27 (45)	32 (53.3)	
Medication, *n* (%)				
OAD	-	47 (78.3)	38 (63.3)	0.071 ^†^
Insulin	-	0 (0)	2 (3.3)	0.154 ^†^
OAD + Insulin	-	13 (21.7)	20 (33.3)	0.152 ^†^
Antihypertensive	-	32 (53.3)	39 (65)	0.194 ^†^
Lipid-lowering	-	30 (50)	31 (51.7)	0.855 ^†^

BMI: body mass index; BP: blood pressure; OAD: oral antidiabetic drugs. Values are expressed as mean ± standard deviation (SD), median (interquartile range: Q1–Q3), or number (percentage), as appropriate. Statistical tests: ^§^ = One-way ANOVA; ^t^ = Independent samples *t*-test; ^†^ = Pearson chi-square test. Post hoc comparisons: ^a^ = HC < T2DM + N (Tukey’s test); ^b^ = HC < T2DM − N and T2DM + N (Games–Howell test).

**Table 2:** Comparison of laboratory parameters among the study groups.

Parameters	Healthy Control (*n* = 60)	T2DM − N *(n =* 60)	T2DM + N *(n =* 60)	*p*
FPG, mg/dL	89 (84–97)	143 (114–208)	196 (154–259)	<0.001 ^¶a^
HOMA-IR	1.4 (1.10–2.10)	4 (2.45–7.06)	6.6 (3.44–11)	<0.001 ^¶b^
HbA1c, %	4.9 (4.75–5.25)	7.9 (6.8–9.15)	8.8 (7.7–10.05)	<0.001 ^¶b^
Creatinine, mg/dL	0.7 (0.63–0.77)	0.79 (0.64–0.95)	0.9 (0.73–1.07)	<0.001 ^¶a^
eGFR, mL/min/1.73 m^2^	102 (95–108)	94 (85–102)	85.11 (69–96)	<0.001 ^¶c^
ALT, U/L	17 (13–21)	16 (13–22)	17.50 (12–21)	0.908 ^¶^
Total Cholesterol, mg/dL	185.17 ± 27.33	196.68 ± 50.68	194.95 ± 47.93	0.293 ^§^
Triglyceride, mg/dL	136 (101–159)	166 (113–222)	150 (118–210)	0.025 ^¶d^
HDLc, mg/dL	45.70 ± 10.89	46.28 ± 9.78	46.50 ± 11.18	0.913 ^§^
LDLc, mg/dL	111.43 ± 25.51	117.3 ± 40.86	114.5 ± 42.05	0.684 ^§^
ESR, mm/h	1.2 (0.8–3)	9 (6–14)	11 (6–16)	<0.001 ^¶b^
CRP, mg/L	0.99 (0.78–2.55)	2.63 (1.22–7.47)	3.2 (1.42–6.78)	<0.001 ^¶b^
Leukocyte, ×10^9^/L	6.46 ± 1.50	8.18 ± 2.34	8.57 ± 2.28	<0.001 ^§e^
UACR, mg/g	2.15 (1.5–5.1)	8.47 (4.6–17.6)	128.79 (57–534)	<0.001 ^¶a^

FPG: fasting plasma glucose; HOMA-IR: homeostasis model assessment of insulin resistance; HbA1c: hemoglobin A1c; eGFR: estimated glomerular filtration rate; ALT: alanine aminotransferase; CRP: C-reactive protein; ESR: erythrocyte sedimentation rate; HDLc: high-density-lipoprotein cholesterol; LDLc: low-density-lipoprotein cholesterol; UACR: urine albumin-creatinine ratio. Values are presented as mean ± standard deviation (SD), median (interquartile range: Q1–Q3), or number (percentage), as appropriate. Statistical tests: ^§^ = One-way ANOVA (Games–Howell post hoc test); ^¶^ = Kruskal–Wallis H test (Dunn’s post hoc correction). Post hoc comparisons: ^a^ = HC < T2DM − N < T2DM + N; ^b^ = HC < T2DM − N and T2DM + N; ^c^ = HC > T2DM − N > T2DM + N; ^d^ = HC < T2DM + N; ^e^ = HC < T2DM − N < T2DM + N.

**Table 3:** Comparison of SIRT2 levels among study groups and subgroups.

Groups	SIRT2 Levels	*p*
Healthy Control ^2,3,4^	6.13 (5.03–8)	<0.001
T2DM − N ^4^	8.53 (6.53–11.45)	<0.001
Microalbuminuria	9.89 (8.3–18.31)	<0.001
Macroalbuminuria	33.19 (7.41–47.72)	<0.001

^2^: *p* < 0.05 vs. T2DM − N group; ^3^: *p* < 0.05 vs. Microalbuminuria group; ^4^: *p* < 0.05 vs. Macroalbuminuria group. HC: healthy control; T2DM − N: type 2 diabetes mellitus without nephropathy; Data are presented as median (interquartile range, IQR). Group comparisons were performed using the Kruskal–Wallis test, followed by Dunn’s post hoc analysis with Bonferroni correction.

**Table 4:** ROC analysis of the combined SIRT2, UACR, and −eGFR model across the three study groups with DeLong comparison against individual biomarkers.

Groups	AUC (95% CI)	Cut-Off	Sensitivity (95% CI)	Specificity (95% CI)	Hosmer– Lemeshow *p*	Brier Score	DeLong Test *p* (Combined vs. Single Biomarker)
HC	0.827 (0.760–0.887)	0.395	0.88 (0.70–0.96)	0.70 (0.63–0.87)	0.0139	0.170	<0.001
T2DM − N	0.656 (0.575–0.738)	0.359	0.82 (0.30–1.00)	0.45 (0.21–0.95)	0.2585	0.205	<0.001
T2DM + N	0.894 (0.849–0.935)	0.221	0.88 (0.77–0.99)	0.76 (0.62–0.88)	0.0630	0.131	<0.001

HC: healthy control; T2DM − N: type 2 diabetes mellitus without nephropathy; T2DM + N: type 2 diabetes mellitus with nephropathy. AUC: area under the curve; CI: confidence interval.

## Data Availability

The data that support the findings of this study are available from the corresponding author upon reasonable request.

## Abbreviations

DN	Diabetic nephropathy
T2DM	Type 2 diabetes mellitus
SIRT2	Sirtuin 2
T2DM − N	Type 2 diabetes mellitus without nephropathy
T2DM + N	Type 2 diabetes mellitus with nephropathy
HC	Healthy control
ESRD	End-stage renal disease
eGFR	Estimated glomerular filtration rate
UACR	Urine albumin–creatinine ratio
BMI	Body mass index
FPG	Fasting plasma glucose
ALT	Alanine aminotransferase
HDLc	High-density lipoprotein cholesterol
LDLc	Low-density lipoprotein cholesterol
CRP	C-reactive protein
ESR	Erythrocyte sedimentation rate
HbA1c	Glycated hemoglobin
HOMA-IR	Homeostasis model assessment of insulin resistance
ELISA	Enzyme-linked immunosorbent assay kit
IQR	Interquartile range
OR	Odds ratio
AUC	Area under the curve
CI	Confidence intervals
